# The Binding of Aripiprazole to Plasma Proteins in Chronic Renal Failure Patients

**DOI:** 10.3390/toxins13110811

**Published:** 2021-11-18

**Authors:** Kenshiro Hirata, Tokunori Ikeda, Hiroshi Watanabe, Toru Maruyama, Motoko Tanaka, Victor Tuan Giam Chuang, Yuji Uchida, Keiki Sakurama, Koji Nishi, Keishi Yamasaki, Masaki Otagiri

**Affiliations:** 1 Faculty of Pharmaceutical Sciences, Sojo University, Ikeda 4-22-1, Nishi-ku, Kumamoto 860-0082, Japan; k-hirata@ph.sojo-u.ac.jp (K.H.); ryousei@ph.sojo-u.ac.jp (T.I.); yuchida1@ph.sojo-u.ac.jp (Y.U.); sakurama-ke@aso-mc.jp (K.S.); knishi@ph.sojo-u.ac.jp (K.N.); 2 Graduate School of Pharmaceutical Sciences, Kumamoto University, Oe-honmachi 5-1, Chuo-ku, Kumamoto 862-0082, Japan; hnabe@kumamoto-u.ac.jp (H.W.); tomaru@kumamoto-u.ac.jp (T.M.); 3 Department of Nephrology, Akebono Clinic, Shirasagi 5-1-1, Minami-ku, Kumamoto 861-4112, Japan; tanaka@matusita-kai.or.jp; 4 School of Pharmacy, Faculty of Health Sciences, Curtin University, GPO Box U1987, Perth, WA 6845, Australia; v.chuang@curtin.edu.au; 5 DDS Research Institute, Sojo University, Ikeda 4-22-1, Nishi-ku, Kumamoto 860-0082, Japan

**Keywords:** uremic toxins, indoxyl sulphate, renal disease, protein binding, aripiprazole

## Abstract

The binding of drugs to plasma protein is frequently altered in certain types of renal diseases. We recently reported on the effects of oxidation and uremic toxins on the binding of aripiprazole (ARP) to human serum albumin. In our continuing investigations, we examined the binding of ARP to plasma pooled from patients with chronic renal dysfunction. We examined the issue of the molecular basis for which factors affect the changes in drug binding that accompany renal failure. The study was based on the statistical relationships between ARP albumin binding and biochemical parameters such as the concentrations of oxidized albumin and uremic toxins. The binding of ARP to plasma from chronic renal patients was significantly lower than healthy volunteers. A rational relationship between the ARP binding rate and the concentration of toxins, including indoxyl sulphate (IS) and p-cresyl sulphate (PCS), was found, particularly for IS. Moreover, multiple regression analyses that involved taking other parameters such as PCS or oxidized albumin ratio to IS into account supports the above hypothesis. In conclusion, the limited data reported in this present study indicates that monitoring IS in the blood is a very important determinant in the dosage plan for the administration of site II drugs such as ARP, if the efficacy of the drug in renal disease is to be considered.

## 1. Introduction

Patients with impaired renal function are prone to demonstrate a high incidence of adverse drug reactions. It is generally assumed that such a high adverse drug reaction rate is mainly due to the accumulation of the drug in the body as the result of a decreased kidney excretion. It is well known that the plasma binding capacity of many drugs is lower in uremic patients than in normal subjects, even after correction for the below-average albumin concentration in these patients. Thus, uremic patients may respond to a drug at a relatively lower total plasma drug concentration than would occur for a nonuremic patient [1].

We recently reported that aripiprazole (ARP), 7-(4-(4-(2,3-dichloropheny)-1-piperazinyl)buthoxy)-3,4-dihydro-2-(1H)-quinolinone, a novel antipsychotic agent, binds strongly to two major plasma proteins, namely, human albumin and α1-acid glycoprotein [2,3]. ARP is effective, especially in long-term use. Its use in hemodialysis patients is expected to be prolonged [4], so it is important to consider the effect of protein binding on its safe use. In particular, for drugs with excellent central transfer properties such as ARP, it is assumed that the effect of an increase in the concentration of the unbound form in the body would have a significant effect on its action.

The present study was undertaken to examine the binding of ARP to plasma of predialysis patients with renal insufficiency and to compare the results with those of normal subjects. In addition, the binding of ARP to human albumin under conditions of oxidation stress in kidney diseases was examined using chloramine-T oxidized human albumin [5]. We also investigated the effects of uremic toxins on the binding of ARP to human albumin. Uremic toxins such as indoxyl sulphate (IS), indole 3-acetic acid, and p-cresyl sulphate (PCS), particularly IS and PCS, inhibited the binding of ARP to albumin [6]. We then attempted to elucidate the molecular mechanism responsible for the interaction of ARP with plasma proteins in kidney diseases, based upon relationships between ARP albumin binding and biochemical parameters such as the plasma concentrations of albumin, oxidized albumin and uremic toxins.

## 2. Results

### 2.1. Patients

Table 1 provides information on the biochemical parameters for blood samples pooled from 24 chronic renal dysfunction patients. The majority of the patients were elderly patients. Serum creatinine (SCr) and blood urea nitrogen (BUN) levels were high. Albumin concentrations were relatively low, with high oxidized albumin ratios. In addition, the concentrations of uremic toxins, including IS and PCS, were significantly elevated compared with healthy volunteers. These clinical data indicate that these patients can be classified as renal dysfunction patients.

### 2.2. Binding of ARP to Plasma

**Figure 1:**
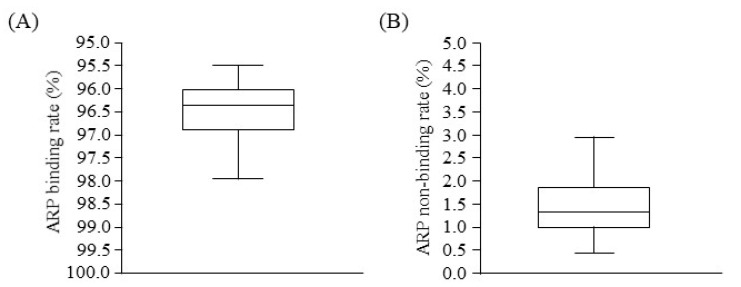
Boxplots in ARP binding (**A**) and non-binding (**B**) rate to plasma proteins of HD patients.

Figure 1 illustrates the binding parameters of ARP to plasma proteins based on an equilibrium dialysis method. Drug binding to plasma proteins was relatively high in hemodialysis (HD) patients. The ARP non-binding rate for plasma from HD patients (median: 1.42, interquartile range (IQR): 1.19–2.01) was significantly higher compared with that for healthy volunteers (median: 0.64, IQR: 0.58–0.67) (Table 1).

### 2.3. Relationship between ARP Binding and Biochemical Parameters

An attempt was made to elucidate the mechanism responsible for the low ARP binding, which may be a characteristic of patients with renal diseases. The Spearman’s correlation coefficient for the ARP non-binding rate and biochemical parameters was examined. As shown in Table 2, we confirmed the existence of a moderate and weak correlation between ARP non-binding and IS (*r* = 0.64, *p* < 0.001) or PCS (*r* = 0.39, *p* = 0.06).

**Figure 2:**
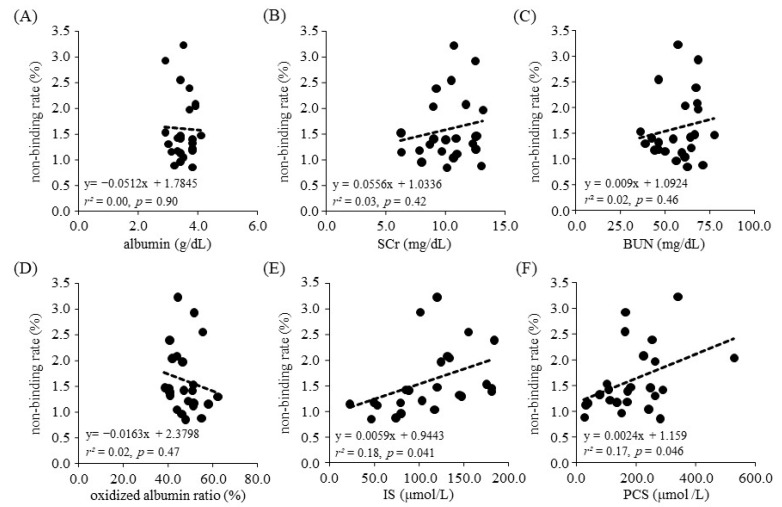
Scatter plot of ARP non-binding rate and each biological parameter. (**A**) albumin, (**B**) SCr, (**C**) BUN, (**D**) oxidized albumin ratio, (**E**) IS, (**F**) PCS.

**Figure 3:**
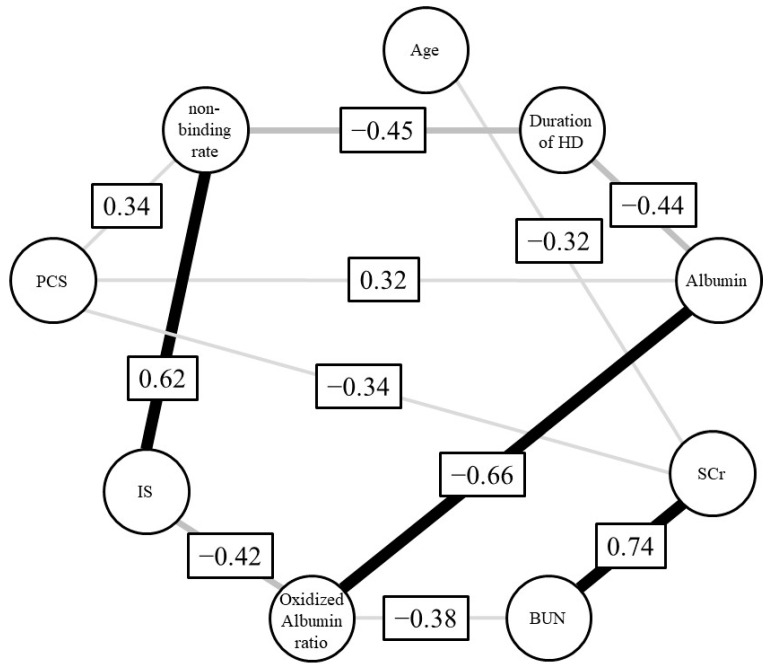
Graphical model of Spearman’s partial correlation coefficient.

We next employed regression analysis to examine the relationship between the ARP non-binding rate and each biological parameter. As a result, no relationship was found between the binding rate for parameters such as albumin concentration, the oxidized albumin ratio, SCr and BUN. In contrast, a relationship between the rate of ARP non-binding and toxins, including the concentration of IS (*r*^2^ = 0.18, *p* = 0.041, 95% confidence interval (CI): 0.00027–0.011) and PCS (*r*^2^ = 0.17, *p* = 0.046, 95%CI: 0.000044–0.0047), was found (Figure 2). Moreover, to investigate the correlation between the ARP non-binding rate and IS or PCS under conditions where the influence of other parameters such as albumin concentration, oxidized albumin ratio, SCr and BUN were excluded, we calculated Spearman’s partial correlation coefficients (Table 3 and Figure 3). The results showed a moderate and weak correlation between the ARP non-binding rate and IS (*r* = 0.62, *p* = 0.008) or PCS (*r* = 0.34, *p* = 0.18), respectively. To confirm these relationships, we used bootstrap testing (*n* = 10,000) and found similar results for both IS (*r* = 0.61, *p* = 0.009) and PCS (*r* = 0.60, *p* = 0.011). These analytical results indicated that uremic toxins such as IS and PCS play an essential and critical role in increasing the ARP non-binding rate in renal diseases.

## 3. Discussion

Diseases can lead to altered concentration and structural change in plasma proteins such as albumin, and accumulation of endogenous substances affect the drug binding to plasma proteins [7,8,9]. Decreased drug binding to plasma protein can occur, especially in renal diseases. We previously reported that ARP, a novel antipsychotic agent, binds to albumin. In this work, we investigated the binding of ARP to plasma proteins in renal diseases using serum samples obtained from patients with renal failure. Biological parameters such as albumin concentrations, SCr, the concentration of toxins, including IS, were initially estimated for the plasma samples used in this study. The binding of ARP to plasma proteins in renal failure was then examined, and the results were compared with the corresponding values for plasma proteins from healthy volunteers. The binding of ARP in the cases of renal failure was reduced significantly, compared with the same values for healthy adults.

To elucidate the reason for this reduced protein binding in renal failure, we conducted statistical analyses for relationships between various biological parameters and ARP binding. A simple analysis of the relationship between ARP binding and each biological parameter indicated that uremic toxins, especially IS, play a critical role in lowering ARP’s binding affinity. Multiple regression results support the conclusion that uremic toxins such as IS significantly affect the binding of drugs to plasma proteins in renal disease. Moreover, the finding that uremic toxins are involved in the reduced drug binding was also supported from the results of a Spearman’s partial correlation coefficient, as shown in Figure 3.

We recently investigated the effects of uremic toxins and albumin oxidation (using chloramine-T) on ARP binding [5,6]. The inhibitory effect of ARP binding by IS (80% increase in the free fraction) was more significant than that by oxidation (40% increase in the free fraction) [5]. The results obtained in this work are in good agreement with our recent results. However, unfortunately, we did not get good results for a multiple regression analysis between ARP binding and those two parameters, namely, IS and the oxidized albumin ratio, and simple relation of the binding and the oxidized albumin ratio was not found. The findings presented herein showing that oxidized albumin did not appear to play an important role in altering the extent of binding by renal failure cannot be fully explained at this time. One of the reasons may be the differences in oxidation conditions. Oxidation in diseased states may be more complicated and different from the simple laboratory oxidation used in the case of chloramine-T. Our previous findings show different effects depending on the oxidation conditions used, for example, oxidation using chloramine-T or metal-catalyzed [10].

In renal failure patients, uremic toxins such as IS, PCS, indole acetic acid, and CMPF (3-carboxy-4-methul-5-propyl-2-furanpropionate) accumulate in serum at concentrations as high as 44.5, 41, 2.4 and 8.8 mg/L [11]. The results of binding experiments indicated that uremic toxins such as IS under conditions of renal disease significantly inhibit the binding of ARP to plasma proteins. Their high concentrations in the blood may have saturated the drug binding site on HSA (ARP and IS all bind to site II) [12]. The reason, however, for why PCS is not significantly correlated with the ARP non-binding rate is not clear. It has been suggested, however, that the spatial orientation of PCS in the binding site may be different from that for IS, it is possible that PCS may inhibit ARP binding differently, especially in these patients. In fact, although PCS is not significantly correlated with the ARP non-binding rate, the result of bootstrap testing was positive. Because our study was limited by the number of available HD patients, it is possible that these results were dissociated. Therefore, it is possible that IS and PCS have different spatial orientations in the binding site, but that PCS may be related to the ARP non-binding rate as well as IS.

There are several limitations associated with this study. First, we did not consider the effects of concomitant drugs. However, since the plasma used in this study was diluted 20-fold, we conclude that it would have a minimal direct impact on the study. The effects of concomitant drugs and the primary metabolite of ARP (i.e., dehydro-ARP) should be investigated using plasma from patients who are actually using ARP. Second, in this study, we used post-dialysis plasma samples. Because of this, we examined the lowest concentrations of unbound uremic toxins. If pre-dialysis blood samples were used, the protein binding of ARP would be expected to be decreased. Differences between the effect using pre- or post-dialysis blood should be examined in the future. Third, we did not determine the ratios of glycation and carbamylation of albumin in the plasma from CKD patients. Further study is needed to clarify whether these post-translational modifications of albumin would be the other factors for the decreased ARP binding in CKD patients.

These findings suggest that IS accumulated in the blood is one of the most important factors for decreasing the drug binding that typically accompanies renal disease. In addition to ARP, caution should also be exercised when the site II drugs commonly prescribed in CKD patients such as ibuprofen, diclofenac or diazepam are concurrently administered.

## 4. Conclusions

In conclusion, the results obtained in the present study indicate the importance of monitoring IS in the blood when developing a dosage plan for the administration of site II drugs (such as ARP) to secure the safety as well as the efficacy of the drug therapy in renal disease.

## 5. Materials and Methods

### 5.1. Chemicals and Materials

Recombinant human albumin was a gift from the Nipro Co. (Shiga, Japan), and albumin was defatted by treatment with activated charcoal at 0 °C in an acidic solution, using a modification of the procedure reported by Chen [13]. IS and indole acetic acid was obtained from Nacalai Tesque Inc. (Kyoto, Japan). PCS was purchased from Tokyo Chemical Industry Co., Ltd. (Tokyo, Japan). All other chemicals were purchased from commercial sources and were of the highest grade available.

Uremic serum was pooled from 24 patients with chronic renal dysfunction who were admitted to the Department of Nephrology at the Akebono Clinic (Kumamoto, Japan). All of the HD patients were receiving regular bicarbonate hemodialysis therapy (4–5 h per person, 3 times per week) using high-flux polysulfonate hollow-fiber dialyzers. Nonuremic pooled serum, prepared from blood samples of 4 healthy men with healthy renal functions. The Institutional Review Board approved the study protocol of Akebono Clinic, and informed consent was obtained in accordance with the Declaration of Helsinki. Blood samples were immediately centrifuged at 4 °C, and plasma aliquots were stored at −80 °C until uses for various analyses. Biochemical parameters were measured at a contract laboratory (SRL, Inc., Tokyo, Japan)

### 5.2. Chromatography of Oxidized Albumin

Plasma albumin was measured by HPLC, as described previously [14]. The HPLC system was composed of an intelligent pump L-6200 equipped with a gradient programmer and an F-1050 fluorescence detector (Hitachi Co., Ltd., Tokyo, Japan). Shodex Asahipak ES-502N column (Showa Denko Co., Ltd., Tokyo, Japan) was used as the stationary phase. The mobile phase consisted of (A) 0.05 mol/L sodium acetate and 0.40 mol/L sodium sulphate mixture (pH 4.85) and (B) 100% ethanol. The gradient step started with 0% of solvent B and finally reached 5% in 30 min. The flow rate was 1.0 mL/min. The excitation/emission wavelengths were 280/340 nm, respectively. From the HPLC profiles, the content of human mercaptalbumin (HMA) and human non-mercaptalbumin (HNA) was estimated by dividing the area of each fraction by the total area corresponding to HSA, as described previously [14].

### 5.3. Chromatography Analyses of Uremic Toxins and Drug

IS and PCS levels were measured by HPLC, as described previously [12]. The HPLC system consisted of an Agilent 1100 series intelligent pump and a fluorescence spectrophotometer. A LiChrosorb RP-18 column (Cica Merk, Tokyo, Japan) was used as the stationary phase. The mobile phase consisted of (A) 100% methanol and (B) 50 mM ammonium formate (pH 4.0) for PCS. Bound material was eluted using a 65–25% B linear gradient from 0-15 min and then 25–65% B from 15-20 min. The flow rate was 1.0 mL/min. PCS was detected using a fluorescence monitor. The excitation/emission wavelengths were 214/306 nm, respectively. The mobile phase consisted of 0.2 M acetate buffer (pH 4.0)/acetonitrile (3:1, *v*/*v*) for IS. The flow rate was 1.0 mL/min. IS was detected using a fluorescence monitor with excitation/emission wavelengths set to 280 and 375 nm, respectively. Plasma ARP concentration was measured by HPLC, as described previously [2]. The HPLC system used in this study consisted of a 655 A-11 pump, 655 A variable wavelength UV monitor (Hitachi Co., Ltd., Tokyo, Japan). The eluent was detected at 210 nm. The stationary phase was a YMC-Pack ODS-AM column (YMC Co., Ltd., Kyoto, Japan) maintained at 40 °C before a 50 μL of sample injection. The mobile phase consisted of (A) 50 mM Sodium dihydrogen phosphate and (B) 100% acetonitrile. The flow rate of the mobile phase was maintained at 1 mL/min. The gradient step started with 30% of solvent B and finally reached 70% in 7 min.

### 5.4. Equilibrium Dialysis

Equilibrium dialysis experiments were carried out using the Rapid Equilibrium Dialysis (RED) Device System (Thermo Fischer Scientific, Waltham, MA, USA). ARP was added to 20-fold diluted plasma at a final concentration of 20 μM for sample preparation, and the sample was incubated for at least 1 h to reach equilibrium. The concentration of ARP was set in the detectable range of free bodies by our HPLC system. The same volume of samples and buffer solutions (67 mM sodium phosphate buffer (pH7.4), 0.3 mL) were inserted into each chamber which was separated by cellulose membranes and gently shaken at 25 °C for 4 h. After equilibrium was achieved, the ARP concentrations in the buffer (C_f_; unbound drug concentration) and plasma protein compartments (20 times dilution of intact plasma: C_b+f_; sum of −80 °C bound and unbound drug concentrations) were measured by HPLC. Bound concentration (C_b_) was calculated by subtracting C_f_ from C_b+f_. Plasma samples were diluted to avoid adsorption to the cells.

### 5.5. Statistical Analysis

This was an observational cross-sectional study. To compare the difference between healthy volunteers and HD patients, the Wilcoxon rank sum test for continuous variables and Fisher’s exact test for categorical variables were performed, respectively. Spearman’s correlation coefficients and a regression model were used to examine the relationship between age, duration of HD, SCr, BUN, albumin, oxidized albumin ratio, IS, PCS and ARP non-binding rate in bivariate. In addition, to investigate the strength of a relationship between an independent variable and a dependent variable, we employed Spearman’s partial correlation coefficients. Partial correlation coefficients identify the correlation between two variables by removing the influence of the third variable [15]. Moreover, bootstrap analysis (*n* = 10,000) for Spearman’s partial correlation coefficients was performed. Analyses were performed using R version 4.0.3 (The R Foundation for Statistical Computing, Vienna, Austria), with statistical significance set at *p* < 0.05.

### 5.6. Ethics

The ethics committee approved this retrospective case-study protocol of the Faculty of Life Sciences, Kumamoto University (Approval No. 1578, Date of Approval: 1 October 2018). All of the subjects provided their written informed consent to participate in this study.

## Figures and Tables

**Table 1:** Comparison of individual values between healthy volunteers and HD patients.

	Healthy Volunteers (*n* = 4)	HD Patients (*n* = 24)	*p*-Value
Age, median (IQR)	24.50 (23.50, 25.50)	78.50 (72.75, 84.00)	0.002
Gender, (Male/Female)	2/2	14/10	1.00
Duration of HD (Year), median (IQR)	-	7.50 (3.00, 12.00)	-
SCr (mg/dL), median (IQR)	0.66 (0.56, 0.74)	10.53 (8.95, 12.31)	<0.001
BUN (mg/dL), median (IQR)	10.10 (9.60, 11.10)	56.65 (45.95, 66.20)	0.002
Albumin (g/dL), median (IQR)	4.30 (4.25, 4.32)	3.45 (3.30, 3.80)	0.002
Oxidized albumin ratio (%), median (IQR)	21.89 (20.05, 24.98)	47.08 (43.10, 50.95)	0.002
IS (mol/L), median (IQR)	5.20 (4.75, 6.02)	118.56 (79.53, 145.57)	<0.001
PCS (mol/L), median (IQR)	10.85 (3.47, 23.06)	170.02 (110.01, 255.26)	0.001
Arp non-binding rate (%), median (IQR)	0.64 (0.58, 0.67)	1.42 (1.19, 2.01)	<0.001

HD = hemodialysis, IQR = interquartile range, SCr = serum creatinine, BUN = blood urea nitrogen, IS = indoxyl sulphate, PCS = p-cresyl sulphate.

**Table 2:** Correlation matrix of Spearman’s correlation coefficient values in bivariate.

	Age	Duration of HD	SCr	BUN	Albumin	Oxidized Albumin Ratio	IS	PCS	Arp Non-Binding Rate
Age									
Duration of HD	0.07 (0.75)								
SCr	−0.32 (0.13)	0.07 (0.74)							
BUN	−0.16 (0.46)	−0.01 (0.97)	0.74 (<0.001)						
Albumin	−0.44 (0.03)	−0.26 (0.22)	0.23 (0.29)	0.23 (0.28)					
Oxidized albumin ratio	0.44 (0.03)	−0.04 (0.87)	−0.27 (0.2)	−0.37 (0.08)	−0.7 (<0.001)				
IS	−0.24 (0.25)	0.07 (0.76)	0.07 (0.73)	0.04 (0.85)	0.16 (0.47)	−0.47 (0.02)			
PCS	−0.17 (0.43)	−0.06 (0.78)	−0.07 (0.74)	0.11 (0.62)	0.40 (0.06)	−0.40 (0.06)	0.33 (0.12)		
Arp non- binding rate	−0.11 (0.59)	−0.26 (0.21)	0.15 (0.48)	0.18 (0.4)	0.10 (0.64)	−0.24 (0.25)	0.64 (0.001)	0.39 (0.06)	

The values given in parentheses indicate *p*-value.

**Table 3:** Correlation matrix of Spearman’s partial correlation coefficient values.

	Age	Duration of HD	SCr	BUN	Albumin	Oxidized Albumin Ratio	IS	PCS	Arp Non-Binding Rate
Age									
Duration of HD	0.11 (0.68)								
SCr	−0.32 (0.21)	0.26 (0.31)							
BUN	0.22 (0.41)	−0.15 (0.57)	0.74 (0.001)						
Albumin	−0.11 (0.67)	−0.44 (0.08)	0.24 (0.35)	−0.22 (0.4)					
Oxidized albumin ratio	0.17 (0.53)	−0.28 (0.29)	0.2 (0.44)	−0.38 (0.14)	−0.66 (0.004)				
IS	−0.11 (0.68)	0.21 (0.43)	0.02 (0.93)	−0.22 (0.39)	−0.18 (0.49)	−0.42 (0.09)			
PCS	−0.06 (0.81)	0.21 (0.42)	−0.34 (0.18)	0.2 (0.43)	0.32 (0.21)	0.01 (0.98)	−0.01 (0.97)		
Arp non- binding rate	0.1 (0.72)	−0.45 (0.07)	0.17 (0.51)	0.06 (0.83)	−0.16 (0.53)	0.05 (0.84)	0.62 (0.008)	0.34 (0.18)	

The values given in parentheses indicate *p*-value.

## Data Availability

Not applicable.
